# Communicating Uncertainty From Limitations in Quality of Evidence to the Public in Written Health Information: Protocol for a Web-Based Randomized Controlled Trial

**DOI:** 10.2196/13425

**Published:** 2019-05-13

**Authors:** Roland Brian Büchter, Cornelia Betsch, Martina Ehrlich, Dennis Fechtelpeter, Ulrich Grouven, Sabine Keller, Regina Meuer, Constanze Rossmann, Andreas Waltering

**Affiliations:** 1 Institute for Quality and Efficiency in Health Care (IQWiG) Cologne Germany; 2 Media and Communication Science University of Erfurt Erfurt Germany

**Keywords:** uncertainty, consumer health information, decision making

## Abstract

**Background:**

Uncertainty is integral to evidence-informed decision making and is of particular importance for preference-sensitive decisions. Communicating uncertainty to patients and the public has long been identified as a goal in the informed and shared decision-making movement. Despite this, there is little quantitative research on how uncertainty in health information is perceived by readers.

**Objective:**

The objective of this study is to design an experiment to examine how different degrees of uncertainty (Q1) and different types of uncertainty (Q2) impact patients’ perception of treatment effectiveness, the body of evidence, text quality, and hypothetical treatment intention. The experiment also examines whether there is an additive effect when multiple sources of uncertainty are communicated (Q3).

**Methods:**

We developed 8 variations of a research summary set in a hypothetical scenario for a treatment decision in the context of tinnitus. These were modified only in the degree of uncertainty relating to the evidence of the presented treatment. We recruited members of the German public from a Web-based research panel and randomized them to one of 8 variations of the research summary to examine the 3 research questions. The trial was only open to the members of the research panel. The outcomes are perception of the effectiveness of the treatment (primary), certainty in the judgement of treatment effectiveness, perception of the body of evidence relating to the treatment, text quality, and decisional intention (secondary). Outcomes were self-assessed. We aimed to recruit 1500 participants to the trial. The recruitment and data collection was fully automated. Ethical approval was waivered by an ethics committee because of the negligible risk to participants.

**Results:**

This protocol is retrospectively published in its original format. In the meantime, the trial was set up and the data collection was completed. Data collection was conducted in May 2018. A total of 1727 eligible panel members were enrolled.

**Conclusions:**

We aim to publish the results in a peer-reviewed journal by the end of 2019. In addition, results will be presented at conferences and disseminated among developers of guidance for the development of evidence-based health information and decision aids.

**Trial Registration:**

German Clinical Trials Register DRKS00015911; https://www.drks.de/drks_web/navigate.do? navigationId=trial.HTML&TRIAL_ID=DRKS00015911 (Archived by WebCite at http://www.webcitation.org/77zyZTGzk)

**International Registered Report Identifier (IRRID):**

DERR1-10.2196/13425

## Introduction

### Background

Uncertainty pervades health care and is integral to evidence-informed decision making. The many layers of uncertainty, however, have hampered a common understanding of the subject. Han et al have developed a helpful taxonomy by identifying various types of uncertainty and classifying them into 3 dimensions [1]:

- Sources of uncertainty: These include, for example, ambiguity arising from conflicting evidence or statistical uncertainty.
- Issues arising from uncertainty: These include difficulties in decision making resulting from scientific uncertainty, for example, regarding treatment effects.
- Their loci, that is, uncertainty may exist in the mind of the patient, the health care provider, or both.

In terms of these dimensions, our experiment examines how communication of scientific uncertainty affects the perception of treatment effectiveness by patients and the public. Helping patients and consumers to understand and deal with uncertainty has been identified as one of the goals of the shared decision making and informed choices movement [2]. Understanding uncertainty is of particular importance for preference-sensitive decisions, that is, when there is a close trade-off between benefits and harms, and patient values and preferences are highly variable. Communicating uncertainty, however, poses many difficulties. Often information providers have to decide which of the many sources of uncertainty are most relevant to patients. Selection is required to prevent information overload, which can prompt people to base their decisions on heuristics rather than evidence [3]. Furthermore, communication of uncertainty may also have detrimental effects, for example, by hampering understanding or decreasing the credibility of the information provider [4,5].

Research on how to communicate uncertainty regarding the benefits and harms of treatments to patients and the public is limited. A systematic review by the Agency for Healthcare Research and Quality identified 8 controlled studies with 9 comparisons, including 6 randomized trials [6]. Of these studies, 4 examined statistical uncertainty, 4 studied different ways of communicating net benefit, and 1 addressed uncertainty arising from the use of a surrogate outcome. These studies were very heterogeneous in terms of context (including cancer screening and treatment decision making), interventions (including written information, drug fact boxes, and multifaceted interventions), and outcomes (including risk perception and decision making).

### Objectives

We are not aware of any studies investigating whether perceptions of uncertainty depend on the degree, type, or amount of uncertainty presented in written health information. Thus, we decided to address the following 3 questions in our study:

1. Degree of uncertainty: Do members of the public perceive treatment effects differently depending on the choice of words used to express the certainty of those treatment effects?
2. Type of uncertainty: Do members of the public interpret uncertain treatment effects differently depending on the type of uncertainty?
3. Number of sources of uncertainty: Is there an additive effect of multiple sources of uncertainty?

We investigated these questions using 8 variations of a written piece of hypothetical consumer health information (research summary) set in a treatment decision scenario in the context of tinnitus. The research summaries were presented to a broad group of members of the German public using a Web-based research panel. Although the study was conducted with Web-based health information, we believe the results will be applicable to all types of written health information, including printed material.

We designed the experiment as a Web-based randomized superiority study, with 8 parallel groups allocated in an equal ratio (between-group design).

## Methods

### Procedures

We recruited members of the public from a Web-based research panel. Panel members had to be at least aged 18 years and able to read and write German. No other inclusion restrictions were applied.

The participants were first provided with a short introduction to the study and an informed consent sheet. We then collected information on age, sex, and educational degree. Participants were then asked to imagine having tinnitus and having unsuccessfully tried several treatments (see Multimedia Appendix 1). They were then randomly presented with one version of different variations of the research summary on the internet. These contained information on the medical condition and a short summary of evidence for a fictitious new tinnitus medication called *Oroxil* (see Multimedia Appendix 1). After presenting participants with the research summaries, we collected data on different outcomes using a questionnaire developed for the purpose of this study. We asked participants to return to the research summary as needed while answering the questions. At the end of the experiment, participants were asked about their profession (medical or nonmedical) and their history of tinnitus (present or not present). Participants were neither aware of the specific research questions nor of the alternative presentations. The original research summaries were written in German and translated into English for this publication.

### Interventions

We chose a treatment scenario in the context of tinnitus and developed 8 variations of the research summary based on our experience and use of language in providing evidence-based health information to consumers through Germany’s statutory health website [7]. In accordance with the objectives of our study, we altered the research summary regarding the degree of uncertainty, the sources of uncertainty, and the number of sources of uncertainty. This resulted in 8 variations, two of which were used in 2 (statistically independent) comparisons (Table 1). An exemplary version of the research summary is provided in the Multimedia Appendix 1.

For the first objective of the study (Q1), we formulated 3 versions of the research summary with different degrees of uncertainty of the treatment effect. One version (A) describes a certain treatment effect and the other (B), a possible, but not certain treatment effect (indication of effect). The third version (B1) is identical to variation B but includes an additional statement on the need for further research. The semantic variations in the degrees of uncertainty of the treatment effect were based on the methods for the assessment of treatment benefits developed by the German Institute for Quality and Efficiency in Health Care (IQWiG) [8].

For the second objective (Q2), we drew on the GRADE (Grading of Recommendations Assessment, Development and Evaluation) framework to categorize different types of uncertainty. According to GRADE, uncertainty can arise from risk of bias, (unexplained) inconsistency, indirectness, imprecision, and other threats to validity, such as publication bias or vested interests [9]. We therefore formulated 3 additional variations of the research summary describing publication bias and vested interests (B2), indirectness (B3), and imprecision (B4). We will also include variation B1 in this comparison.

For the third objective of the study (Q3), we developed 2 further variations that contained a combination of 2 or 3 sources of uncertainty (B42 and B432). A variation including only 1 source of uncertainty (B4) is included in this comparison.

**Table 1:** Variations of the research summary used to examine the 3 overarching research questions (translated from German).

Variations for research objective and identifier		Version	Variation in text
**Q1. Degree of uncertainty**			
	A	Effect shown	Studies show that Oroxil can reduce tinnitus.
	B	Indication of effect	Studies indicate that Oroxil may reduce tinnitus.
	B1	Indication of effect with general explanation	Studies indicate that Oroxil may reduce tinnitus. (...) The pros and cons of Oroxil cannot be fully judged, however. This requires further research.
**Q2: Type of uncertainty**			
	B1	Indication of effect with general explanation	As above
	B2	Publication bias/vested interests	Studies indicate that Oroxil may reduce tinnitus. (...) The pros and cons of Oroxil cannot be fully judged, however. The reason for this is that the company that developed the drug has not published all the studies on Oroxil.
	B3	Indirectness (population)	Studies indicate that Oroxil may reduce tinnitus. (...) The pros and cons of Oroxil cannot be fully judged, however. The reason for this is that the people who took part in the study were exposed to loud noises at work. It is uncertain whether the results also apply to other people.
	B4	Imprecision (small sample size)	Studies indicate that Oroxil may reduce tinnitus. (...) The pros and cons of Oroxil cannot be fully judged, however. The reason for this is that only a small number of people took part in the studies.
**Q3: Multiple sources of uncertainty**			
	B4	Imprecision (small sample size)	As above
	B42	Publication bias/vested interests and imprecision	Studies indicate that Oroxil may reduce tinnitus. (...) The pros and cons of Oroxil cannot be fully judged, however. The reason for this is that only a small number of people took part in the studies. Furthermore, the company that developed the drug has not published all the studies on Oroxil.
	B432	Publication bias/vested interests and imprecision and indirectness	Studies indicate that Oroxil may ease tinnitus. (...) The pros and cons of Oroxil cannot be fully judged, however. The reason for this is that the studies were small. Furthermore, the people who took part in the studies were exposed to loud noises at work. It is uncertain whether the results also apply to other people. Finally, the company that developed the drug has not published all studies on Oroxil

### Outcomes

Our primary outcome is the perception of treatment effectiveness. The secondary outcomes are subjective certainty in the judgement of treatment effectiveness, perception of the body of evidence, hypothetical treatment intention, and perception of text quality.

The perception of treatment effectiveness was measured with 1 item on an ordinal scale (*How do you judge the effectiveness of Oroxil?*) with 5 possible answers: (a) It is proven that Oroxil can help; (b) Oroxil may possibly help; (c) It is unclear, whether Oroxil helps; (d) Oroxil may not help; and (e) Oroxil definitely does not help.

Subjective certainty in the judgement of treatment effectiveness was measured on a 5-point Likert scale, ranging from *not certain at all* to *very certain*. As this relates to the first question on perceptions of treatment effectiveness, data on this item were gathered immediately after answering the first question.

The perception of the body of evidence was measured with a 6-item semantic differential (Cronbach alpha=.81), with each item measured on a 5-point Likert scale. Participants were asked to rate the body of evidence as follows:

- Certain to uncertain
- Reliable to unreliable
- Valid to not valid
- Generalizable to not generalizable
- Excellent to poor
- Trustworthy to untrustworthy

The hypothetical treatment intention was measured using 1 item (*How would you decide?*) measured on a 5-point Likert scale with 2 poles: (a) definitely not take Oroxil and (b) definitely take Oroxil.

The perception of text quality was measured with a 9-item semantic differential (Cronbach alpha=.81) based on previous literature [10,11]. The construct included the following items measured on a 5-point Likert scale:

- Interesting to uninteresting
- Balanced to 1-sided
- Comprehensible to incomprehensible
- Credible to incredible
- Clear to unclear
- Well done to not well done
- Professional to unprofessional
- Appealing to not appealing
- Respectable to not respectable

### Mediators (Explorative)

We collected data on the following possible mediating variables for the purpose of explorative analyses:

- Decisional conflict (German version of the uncertainty subscale of the decisional conflict scale, Cronbach alpha=.76) [12]
- Perceived knowledge about the treatment measured on a visual analogue scale ranging from 0 for *no knowledge* to 100 for *all possible knowledge* [13]
- Perceived sufficiency of knowledge about the treatment for decision making, measured on an ordinal scale with 3 possible answers (more knowledge, the amount of knowledge provided, and less knowledge)
- Perception of the credibility of the information provider (based on previous scales, Cronbach alpha=.93) [14,15]

### Moderators (Explorative)

We collected data on the following possible moderator variables, again, for the purpose of explorative analyses:

- Sex
- Age
- Educational degree based on the German school system (none/basic secondary/higher secondary/general entry qualification for university/university degree)
- Subjective health literacy (using the German version of the Brief Health Literacy Screening Tool (known as BRIEF), Cronbach alpha=.76) [16]
- Numeracy (using the 1-item version of the Berlin Numeracy Test) [17]
- Objective subscale of the perceived uncertainty of scientific evidence scale (Cronbach alpha=.76) [18]
- Medical degree or profession (yes/no)
- Previous experience with tinnitus (history of tinnitus/currently symptomatic/never present)

We piloted a paper-and-pencil version of the questionnaire with 2 versions of the research summary in a convenience sample of 40 students to test the reliability of the constructs, comprehensibility of instructions, the stimuli, and the questions. The reliability of the constructs was good to very good as reported above (Cronbach alpha ranging from .76 to .93). On the basis of the pretest, we omitted 2 items from the pilot questionnaire for the outcome of perceived text quality to increase reliability. We also amended the instructions to improve comprehensibility.

### Statistical Analysis

We will present demographic characteristics of the sample using frequencies, in case of categorical data, and measures of location (mean and median) and variation (SD, interquartile ranges [IQRs], and ranges), in case of continuous data.

We will treat the primary outcome variable as an ordinal scale with 5 possible values, where higher values indicate an increase in the perception of effectiveness (5=it has been proven that the treatment can help to 1=treatment definitely cannot help). We will present data as medians, IQRs, and ranges for each group. We will also present means and SDs, as well as the proportions for each possible answer in descriptive tables. This will also help to establish the practical relevance of the results.

For the secondary outcomes *perception of the body of evidence* and *text quality*, we will combine the items of each of the scales into 1 index by averaging their values, where a higher value will indicate better perception of the body of evidence or text quality.

For our confirmatory statistical analyses of the primary and secondary outcomes, we will conduct Kruskall-Wallis tests to test for overall differences between the groups within each of our 3 primary study questions. We chose to use a nonparametric test to account for the types of scales used (ordinal scaling or unequal differences between items).

In case of statistical significance, we will conduct a multiple testing procedure to perform pairwise comparisons within the groups by means of the Dwass-Steel-Critchlow-Fligner multiple comparison analysis, which is based on pairwise 2-sample Wilcoxon comparisons. All comparisons between groups across the 3 overarching study questions will be considered explorative (eg, A vs B432).

We will conduct sensitivity analyses by means of an analysis of variance (ANOVA) to test for the overall differences between the groups within each of our 3 overarching study questions. In case of statistical significance, we will conduct pairwise comparisons by means of Tukey honestly significant difference procedure. We will inspect data to ensure that they meet distributional assumptions (normality and equal variance) before applying statistical tests.

Statistical analyses will be conducted in SAS version 9.4 (SAS Institute Inc). All statistical tests will be 2-sided and performed using a 5% significance level. Where applicable, differences in means between groups will be presented together with a 95% CI. All analyses will be conducted on an intention-to-treat basis. Explorative analyses based on potential moderators and mediators are not predetermined.

As we will collect outcome data immediately after the presentation of the research summaries and panel members need to finish the questionnaire to receive an incentive, we have no major concerns regarding missing data. Furthermore, we assume that any dropouts will be likely to be missing at random, as we believe it is unlikely that the intervention has an influence on the responses to the questionnaire. Thus, we do not plan to employ any imputation methods. In case of missing data, we will present this information descriptively.

As the experiment is Web-based and participants come from a panel that provides incentives for participation, there is a risk that some participants only participate to collect their incentive and do not provide valid answers. As a measure of quality assurance, we will exclude data from participants who answer all questions in less than 2 min, spend less than 20 seconds on the page displaying the research summary, and spend less than 1.5 min between reading the research summary and completing the questionnaire (so called speeders). These time limits were determined by a priori test readings. We will also exclude participants who provide all answers in the same column for the matrix questions, that is, when more than 1 item is displayed on the screen (so called straightliners).

### Sample Size Calculation

We based the sample size calculations for all 3 research questions on the following considerations and assumptions. We used the comparison of 4 groups (which corresponds to the second study question) as a basis and proceeded from a 1-way ANOVA with equal sample sizes in each group. We assumed a significance level of 5% and a statistical power of 90%. We decided to assume an effect size of *F*=0.15 for the primary outcome (confirmatory analysis), where *F* denotes the ratio of the SD of the group means and the common SD within each group. This decision was made based on a pretest of 2 of the research summaries, where we found small effect sizes in the range of up to *F*=0.3, depending on the outcome variable. According to Cohen, the chosen value of *F* lies between a small (*F*=0.10) and a medium (*F*=0.25) effect size [19]. Sample size calculations were conducted with nQuery version 3.0 (Statistical Solutions Ltd). This resulted in a number of 159 participants in each group. As the primary analysis is a nonparametric test, we added 15% according to a general rule of thumb [20]. To allow some leeway, we decided to randomize a total of 1500 participants, equaling an average of 187.5 participants per group.

### Data Collection and Allocation Procedure

The data collection was run by the Survey Centre Bonn (uzbonn—Gesellschaft für empirische Sozialforschung und Evaluation), a spinout company of the Center for Evaluation and Methods at the University of Bonn. UNICOM Intelligence (formerly IBM SPSS Data Collection) was used for data collection (UNICOM Systems, Inc). This software uses Microsoft’s .NET Framework 4.0 random generator to generate random numbers to allocate the participants to the research summaries. A quota was used to ensure equal representation of different age groups and sexes. Once a quota cell was full, enrollment for this quota was closed. Thus, allocation happened after panel members answered demographic questions and were computer-checked for eligibility. As the experiment was entirely Web-based, the allocation sequence was concealed from investigators and data collectors. The data analyses will be conducted by a statistician from the Medical Biometry Department at IQWiG.

### Ethics and Dissemination

The study was presented to the ethics committee at the University of Erfurt (EV-20180921). The committee decided that the research was exempt from the requirement of ethical approval because of its negligible risk to participants and as only nonidentifiable data were collected. The study results will be disseminated via publication and conference presentations.

## Results

The trial was set up between February and April 2018. Data collection was completed in May 2018. Recruitment and data collection were Web-based. First, a website, only accessible via a link available to invited panel members, was set up (password protected site). Font and color use matched the appearance of the national German consumer health website [7]. Then, 6 of the authors (RBB, ME, DF, UG, RM, and AW) read and reread the recruitment texts and the research summaries. The same authors also tested the questionnaire used for data collection. After 2 rounds of debugging, the website and questionnaire were finalized and data collection was started.

Participants were recruited by the Survey Centre Bonn from a Web-based panel of members of the German public. The panel was provided by the online access panel provider Bilendi. Participation was only permitted with use of a desktop computer.

Participants were first informed about the general purpose of the study (to study the perception of health information), the duration required for answering the questionnaire, and the use of data. Only anonymous data were collected. The outcome data were collected over 10 consecutive screens. Participants were able to move forward and backward between the screens. However, once they completed the data collection for the primary and secondary outcomes, moving backward to that section of the questionnaire was not possible anymore. The order of items in the multiitem outcomes was presented in random order. We used soft reminders to encourage participants to answer all questions, that is, the participants were asked to complete unanswered questions before proceeding but were not obliged to answer them. The only mandatory questions were regarding age and sex, as this information was needed to check eligibility. Repeated participation by the same panel member was prevented by the use of a password encrypted access link, which was provided to participants via email.

In total, 2099 invited panel members were assessed for eligibility and 1727 were randomized to 1 of the 8 groups.

## Discussion

We aim to publish the results in a peer-reviewed journal by the end of 2019. In addition, results will be presented at conferences and disseminated among developers of guidance for the development of evidence-based health information and decision aids.

## Appendix

Multimedia Appendix 1 Introduction text and exemplary research summary.

## Abbreviations

ANOVA: analysis of variance
GRADE: Grading of Recommendations Assessment, Development and Evaluation
IQRs: interquartile ranges
IQWiG: the German Institute for Quality and Efficiency in Health Care
