# Supplementary material for: Communicating Uncertainty From Limitations in Quality of Evidence to the Public in Written Health Information: Protocol for a Web-Based Randomized Controlled Trial
Source: JMIR Res Protoc. 2019 May 13;8(5):e13425. doi: 10.2196/13425 (PMC6535974; doi:10.2196/13425)
Supplement: Multimedia Appendix 1 [file resprot_v8i5e13425_app1.pdf]

Multimedia Appendix 1

Introduction text

Please imagine the following situation:

You have had tinnitus for a while now. Tinnitus leads to bothersome noises in the ear such as ringing, hissing, buzzing or roaring. The symptoms can last for months or years. In many people, its causes remain unclear, which makes tinnitus difficult to treat.

You have tried a number of different treatments, none of which has helped. Recently, a new drug has appeared on the market. You decide to inform yourself about the benefits of this drug. We ask you to read the following information carefully and answer a few questions regarding the text.

Exemplary research summary (Variation A):

Treatments for tinnitus

Several treatments are offered and used for tinnitus. None of them is particularly effective, however. A new drug called Oroxil has recently become available.

Oroxil improves the blood flow to the inner ear. This is hoped to reduce tinnitus noises or get rid of them completely. Oroxil is taken as a tablet once a day.

What are the pros and cons of Oroxil?

Studies show that Oroxil can reduce tinnitus symptoms. In these studies, one half of the participants took the drug, the other half a placebo (fake drug). After six months, the groups were compared. The result:

- Without treatment, ear noises significantly improved in about 20 out of 100 people.
- With treatment, ear noises significantly improved in about 25 out of 100 people.

In other words, Oroxil improved tinnitus symptoms significantly in an extra 5 out of 100 people after 6 months.

The drug lead to minor side effects such as occasional dizziness or tiredness in 3 out of 100 people. No severe side effects occurred.
